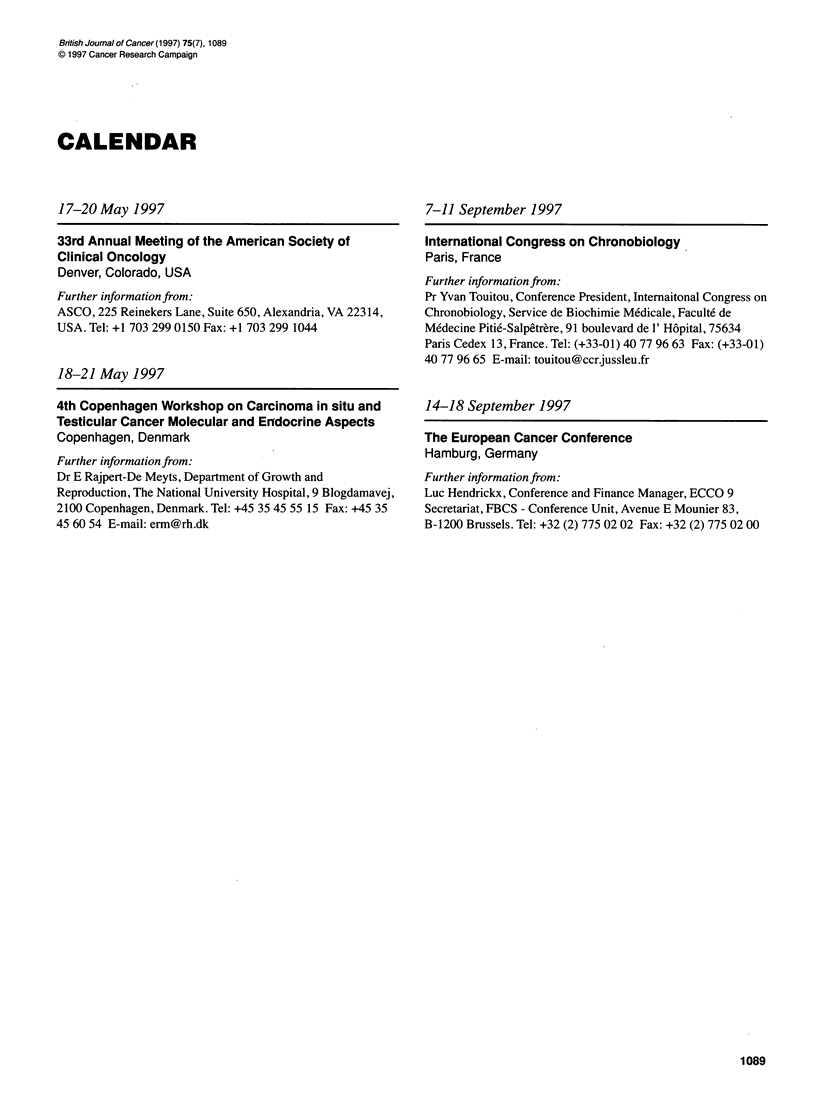# Calendar

**Published:** 1997

**Authors:** 


British Journal of Cancer (1997) 75(7), 1089
© 1997 Cancer Research Campaign

### CALENDAR

*17–20 May 1997*

**33rd Annual Meeting of the American Society of Clinical Oncology**
Denver, Colorado, USA

*Further information from:*
ASCO, 225 Reinekers Lane, Suite 650, Alexandria, VA 22314, USA. Tel: +1 703 299 0150 Fax: +1 703 299 1044

*18–21 May 1997*

**4th Copenhagen Workshop on Carcinoma in situ and Testicular Cancer Molecular and Endocrine Aspects**
Copenhagen, Denmark

*Further information from:*
Dr E Rajpert-De Meyts, Department of Growth and Reproduction, The National University Hospital, 9 Blogdamavej, 2100 Copenhagen, Denmark. Tel: +45 35 45 55 15 Fax: +45 35 45 60 54 E-mail: erm@rh.dk

*7–11 September 1997*

**International Congress on Chronobiology**
Paris, France

*Further information from:*
Pr Yvan Touitou, Conference President, Internaitonal Congress on Chronobiology, Service de Biochimie Médicale, Faculté de Médecine Pitié-Salpêtrère, 91 boulevard de l' Hôpital, 75634 Paris Cedex 13, France. Tel: (+33-01) 40 77 96 63 Fax: (+33-01) 40 77 96 65 E-mail: touitou@ccr.jussleu.fr

*14–18 September 1997*

**The European Cancer Conference**
Hamburg, Germany

*Further information from:*
Luc Hendrickx, Conference and Finance Manager, ECCO 9 Secretariat, FBCS - Conference Unit, Avenue E Mounier 83, B-1200 Brussels. Tel: +32 (2) 775 02 02 Fax: +32 (2) 775 02 00